# Red blood cells in proliferative kidney disease—rainbow trout (*Oncorhynchus mykiss*) infected by *Tetracapsuloides bryosalmonae* harbor IgM^+^ red blood cells

**DOI:** 10.3389/fimmu.2023.1041325

**Published:** 2023-02-15

**Authors:** Justin T. H. Chan, Amparo Picard-Sánchez, Jovana Majstorović, Alexander Rebl, Dirk Koczan, Filip Dyčka, Astrid S. Holzer, Tomáš Korytář

**Affiliations:** ^1^ Laboratory of Fish Protistology, Institute of Parasitology, Biology Centre, Czech Academy of Sciences, České Budějovice, Czechia; ^2^ Faculty of Science, University of South Bohemia, České Budějovice, Czechia; ^3^ Fish Genetics Unit, Institute of Genome Biology, Research Institute for Farm Animal Biology, Dummerstorf, Germany; ^4^ Core Facility for Microarray Analysis, Institute for Immunology, Rostock University Medical Centre, Rostock, Germany; ^5^ Division of Fish Health, Veterinary University of Vienna, Vienna, Austria; ^6^ South Bohemian Research Centre of Aquaculture and Biodiversity of Hydrocenoses, Institute of Aquaculture and Protection of Waters, Faculty of Fisheries and Protection of Waters, University of South Bohemia, České Budějovice, Czechia

**Keywords:** proliferative kidney disease (PKD), erythrocytes, antibody, renal disease, bony fish, anemia, innate immunity

## Abstract

The myxozoan parasite *Tetracapsuloides bryosalmonae* is the causative agent of proliferative kidney disease (PKD)—a disease of salmonid fishes, notably of the commercially farmed rainbow trout *Oncorhynchus mykiss*. Both wild and farmed salmonids are threatened by this virulent/deadly disease, a chronic immunopathology characterized by massive lymphocyte proliferation and hyperplasia, which manifests as swollen kidneys in susceptible hosts. Studying the immune response towards the parasite helps us understand the causes and consequences of PKD. While examining the B cell population during a seasonal outbreak of PKD, we unexpectedly detected the B cell marker immunoglobulin M (IgM) on red blood cells (RBCs) of infected farmed rainbow trout. Here, we studied the nature of this IgM and this IgM^+^ cell population. We verified the presence of surface IgM *via* parallel approaches: flow cytometry, microscopy, and mass spectrometry. The levels of surface IgM (allowing complete resolution of IgM^-^ RBCs from IgM^+^ RBCs) and frequency of IgM^+^ RBCs (with up to 99% of RBCs being positive) have not been described before in healthy fishes nor those suffering from disease. To assess the influence of the disease on these cells, we profiled the transcriptomes of teleost RBCs in health and disease. Compared to RBCs originating from healthy fish, PKD fundamentally altered RBCs in their metabolism, adhesion, and innate immune response to inflammation. In summary, RBCs play a larger role in host immunity than previously appreciated. Specifically, our findings indicate that the nucleated RBCs of rainbow trout interact with host IgM and contribute to the immune response in PKD.

## 1 Introduction

Myxozoans (phylum Cnidaria) are parasites that infect a wide range of teleost fishes. Bony fishes serve as alternate hosts for this group of evolutionarily reduced parasites. The longstanding relationship between and coevolution of myxozoans and teleosts have driven their diversification (1). Furthermore, this continuing arms race has led to myxozoans adopting evasion mechanisms and teleost fishes developing immune defense mechanisms (2). We must study the host-parasite relationship to understand how we can confer protection against myxozoans and limit pathologies resulting from infection.

Proliferative kidney disease (PKD) is caused by *Tetracapsuloides bryosalmonae* (Myxozoa: Malacosporea), a cnidarian endoparasite infecting salmonid fishes. It threatens wild and farmed salmonids in North America and Europe (3). Following a two-host life cycle, disease outbreaks occur annually between late spring and early autumn (4). During this period, invertebrate bryozoans release spores which infect fish *via* the gills or skin (5, 6). Parasites then circulate preferentially to organs such as the kidney, spleen, and liver, where they proliferate. The infection is characterized by lymphocytic hyperplasia in the kidneys (7), spleen swelling, and anemia.

Rainbow trout *Oncorhynchus mykiss* is an irregular, dead-end host for *T. bryosalmonae* and only occasionally survives severe infections with mortality rates reaching up to 95% in hatchery-reared fish. In contrast, the native host, the brown trout, is more resilient, and enables spore formation and parasite transmission. Following an initial exposure to the parasite, surviving fish develop immunological memory, produce adaptive immune antibody-secreting B cells, and are protected from subsequent re-exposure to *T. bryosalmonae* (8).

Research into PKD and other myxozoan models has identified several common features in the immune responses towards this parasitic clade (2). For instance, *T. bryosalmonae* (9), *Sphaerospora molnari* (10), *Ceratonova shasta* (11), and *Enteromyxum leei* (12, 13) myxozoan infections all induce transcriptional signatures that include increased *il10* expression alongside observations of B lymphocyte proliferation and hypergammaglobulinemia.

In PKD, the antibody repertoire is altered with hallmarks of polyreactivity which suggests that only a portion of secreted antibodies are parasite-specific (9). As the teleost head kidney serves as an erythropoietic, lymphopoietic, and lymphoid organ, not only B cells but other immune cell populations and components of the immune response are also affected by the parasite, with several studies demonstrating upregulation of B and T (helper) cell markers of differentiation following infection (8, 9, 14).

While studying the B cell population of rainbow trout infected by *T. bryosalmonae* and that subsequently developed PKD (hereafter, referred to as “fish with PKD”, “diseased fish”, or “infected fish”), we unexpectedly detected IgM^+^ red blood cells (RBCs). As a B cell lineage marker, IgM is exclusively expressed by B cells (15). IgM is present on B cell surfaces and also secreted to bind and to tag pathogens for destruction. Since these do not apply to RBCs, it is possible that PKD elicits antibodies binding to antigens on the RBC surface or that fish RBCs capture antibody-antigen immune complexes as they do in mammals.

RBCs are often overlooked and their activities reduced to oxygen transport. However, their roles in immunity and in disease should not be neglected, especially in teleost fishes whose RBCs are nucleated. Evidence suggests that fish RBCs respond to immune stimuli and express antimicrobial peptides and cytokines (16–19). In addition to their innate immune capacities, the present study provides evidence that RBCs also interact with IgM.

## 2 Results

### 2.1 IgM^+^ RBCs in the blood of rainbow trout with PKD

During August 2020, between the 8^th^ and 9^th^ week of summer, we visited a commercial fish farm in the South Bohemian region of the Czech Republic. We selected fish sharing both the same pond and external signs of the disease such as visible abdominal distension, and lethargy. Upon dissection, we observed clinical and pathological signs including pale gills, swollen spleens and kidneys (**Supplementary Figure 1A**). All naturally infected fish sampled had grade 3-4 renal swelling indices, based on the kidney swelling grading system established by Clifton-Hadley et al. (20) and characterized by kidneys appearing severalfold the volume of healthy kidneys along with abnormal folds, and spots of grey and pink on capsule surfaces (**Supplementary Figure 1A**). To detect *T. bryosalmonae*, we stained kidney imprints using eosin-methylene blue and identified extrasporogonic stages of the parasite (**Supplementary Figure 1B**). None of these signs nor the parasite were detectable in control specific pathogen-free (SPF) fish (hereafter, referred to as the “control” group).

In an initial attempt to study trout IgM^+^ B lymphocytes in PKD, we applied a monoclonal anti-trout IgM antibody (mAb 1.14) (21, 22) towards enrichment of this population in spleen and anterior kidney. This antibody binds to a subpopulation of lymphocytes (21, 22) and we confirmed its specificity *via* an IgG1 isotype control (**Supplementary Figure 2**). In one representative individual, as expected, leukocytes were enriched to 91.1% and 97% IgM^+^ cells in the positive fractions of spleen and anterior kidney, respectively (**Supplementary Figure 3**).

**Figure 1:**
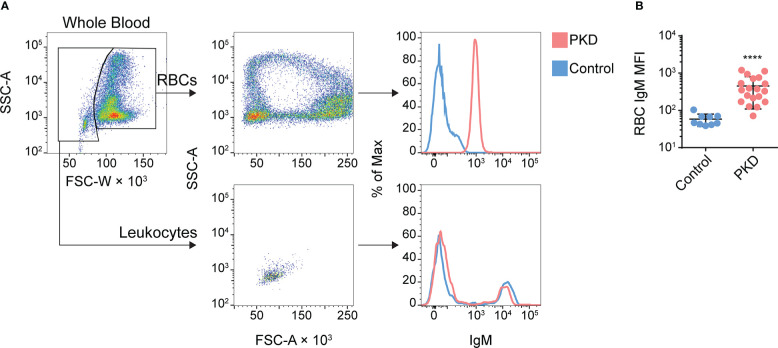
IgM^+^ red blood cells (RBCs) are detectable and abundant in the circulation of rainbow trout with proliferative kidney disease (PKD). **(A)** Here, we give an example of the gating strategy we used for discrimination of RBCs from non-RBCs/leukocytes RBCs can be largely identified by their distinct side scatter area (SSC-A) versus forward scatter width (FSC-W) profile (top left panel). Better resolution can be achieved *via* DiOC6 staining (**Supplementary Figure 4**). We present the result in the middle column: a ring profile for RBCs (top) and an FSC- and SSC-low population characteristic of lymphocytes (bottom). In the rightmost panels, we overlayed histograms of RBCs (top) or lymphocytes (bottom) from infected (red) or control fish (blue) after staining with mAb 1.14. The entire population of RBCs shifts clearly away from the population of control RBCs whereas leukocytes from the same two representative fish show no such difference. **(B)** Summary of the surface IgM phenotype for the entire cohort of control and PKD fish, with each point representing the gated RBCs from one individual and this population’s level of surface IgM. The mean +/- SD of the MFI of mAb 1.14 staining was plotted. The difference between the “Control” and “PKD” group was significant by the Mann-Whitney U test, ****P < 0.0001; n = 9 for control specific pathogen-free fish and n = 19 for the PKD cohort.

Unexpectedly, the flow cytometric analysis of enriched cells revealed a high proportion of RBCs which suggests the presence of IgM on the surface of RBCs (**Supplementary Figure 3**). To further confirm this observation, we next stained whole blood with mAb 1.14 and performed flow cytometry analyses (full gating strategy presented in **Supplementary Figure 4**). Alongside the B cells (**Figure 1A**, bottom row), we detected IgM^+^ RBCs in the diseased fish (**Figure 1A**, top row) but not in control fish. Remarkably, the IgM^-^ and IgM^+^ leukocytes (serving as internal negative and positive controls respectively) overlapped in IgM expression between control fish and fish with PKD (**Figure 1A**, bottom right histogram and **Supplementary Figure 5**). However, the whole population of RBCs from the infected fish clearly shifted away from the control RBCs in levels of IgM (**Figure 1A**, top right histogram). Since we do not fix nor permeabilize cells, the IgM we detected is on the surface of the RBCs. This phenomenon was observed repeatedly in at least 17 other fish with detectable *T. bryosalmonae* PKD stages and/or displaying signs of PKD (**Figure 1B**). Although variable between fish, on average, we observed an almost eight-fold increase in mAb 1.14 staining of RBCs from infected fish compared to control fish tested in parallel (**Figure 1B**). Importantly, the amount of detectable IgM on the surface of RBCs did not reach that of IgM^+^ B lymphocytes which displayed almost 10 times more IgM than the RBCs (**Figure 1A**).

**Figure 2:**
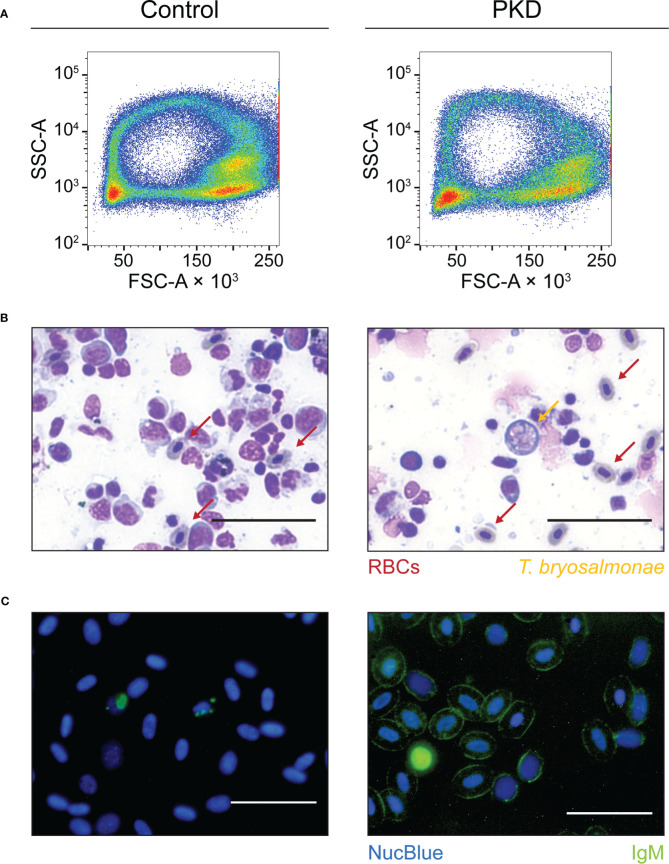
The red blood cells (RBCs) from control fish and infected fish are comparable in morphology and complexity. The labels at the top of the figure apply to the whole figure, not only to **Figure 2A**. They indicate that images or plots shown on the left column originate from control fish and that those from the right column originate from infected fish (PKD). **(A)** As an initial population level study of the size and density/granularity of the RBC population (every cell of a population represented by one event or “dot”), we profiled the forward scatter (FSC) and side scatter (SSC) respectively of RBCs from control and infected fish (PKD). The RBC population of both groups are comparable and representative of measurements taken from all fish studied. Cell clusters are apparent from the colors red and yellow indicating higher density of events or cells at those locations. The RBCs shown from the individual with PKD are over 95% IgM^+^ as determined by flow cytometry. **(B)** An eosin-methylene blue stain also reveals the identical morphology of RBCs from the two groups (indicated by red arrows) alongside extrasporogonic *T. bryosalmonae* indicated by an orange arrow. These stains are from anterior kidney imprints in which IgM^+^ RBCs are also abundant (**Supplementary Figure 2**). Furthermore, **(C)** we independently verified the surface IgM phenomenon on RBCs from control fish and fish with PKD by fluorescence microscopy of whole blood stained with mAb 1.14 (green) and the NucBlue Live ReadyProbes Reagent (blue) that also reveals the regular morphology of IgM^+^ RBCs. All scale bars represent 50 μm.

We did not observe any morphological difference between IgM^-^ and IgM^+^ RBCs from control fish and infected fish, respectively. Flow cytometric forward scatter and side scatter estimate the size and granularity/density of cells. Analyzing these two scatter parameters, the flow cytometric profile of RBCs from representative control fish and fish with PKD were comparable, with the same “ring” profile typical of non-spherical RBCs (**Figure 2A**). Both groups harbor comparable clusters of RBCs (**Figure 2A**) indicated by the color red which reflects the density/concentration of cells in the plots. This applies to the RBCs of every fish studied (data not shown). By microscopy, nuclear and cytoplasmic staining using eosin-methylene blue also did not reveal any differences between RBCs from the two groups (**Figure 2B**). RBCs from fish with PKD maintain their oval shape and morphology. In parallel, the presence of surface IgM was confirmed using fluorescence microscopy (**Figure 2C**). We detected IgM on RBCs (**Figure 2C**, right image) and this observation supports those obtained by flow cytometry (**Figure 1A**, **Supplementary Figures 4, 5**). In contrast, in healthy fish, we did not detect IgM on RBCs (**Figure 2C**, left image). In summary, we observed that RBCs with surface IgM are a signature of PKD in rainbow trout. The RBCs of infected fish did not have visible changes in morphology.

### 2.2 PKD induces hypergammaglobulinemia but not autoreactive antibodies against RBCs

**Figure 3:**
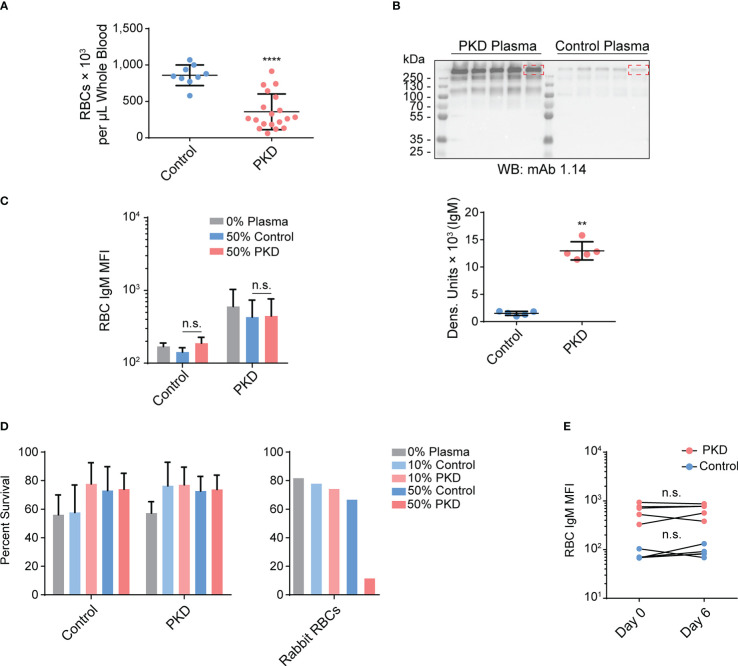
Despite anemia and IgM hypergammaglobulinemia in infected fish with proliferative kidney disorder (PKD), the IgM from fish with PKD is neither autoreactive nor hemolytic. **(A)** *Via* flow cytometry, we quantified the number of red blood cells (RBCs) per μL of whole blood and determined that fish with PKD were anemic. We plotted mean +/- SD of the number of RBCs per μL of whole blood. The difference between the control and PKD groups was determined to be significant by the Mann-Whitney U test; n = 9 for control fish and n = 19 for the PKD cohort. **(B)** mAb 1.14 immunoblotting on plasma (diluted to 3.75%) from randomly selected fish in both the PKD and control groups. We performed densitometry using the dominant IgM band (indicated by dashed red rectangles) that was visible in both groups. The Mann-Whitney U test determined that the amount of circulating IgM was significantly higher in the plasma of PKD fish. RBCs from individuals in control or PKD groups (n = 5, in each group) were incubated in 10% or 50% plasma pooled from either control fish or from PKD fish. We measured surface IgM levels **(C)** and percent surviving propidium iodide-negative cells **(D)** among RBCs after 20 hours of culture. Any changes in either parameter were calculated using a two-way repeated measures ANOVA. A *post hoc* Tukey’s multiple comparisons test revealed that there were no significant differences in surface IgM levels nor cell viability between cells treated with identical concentrations of pooled experimental plasma from PKD and control animals. However, when we used rabbit RBCs as target cells instead, the 50% PKD rainbow trout plasma was potent and lysed nearly all RBCs unlike the control plasma. **(E)** To further support that IgM on the surfaces of RBCs are not bound to cognate RBC antigens, the phenotype was monitored after six days of *in vitro* culture. Just like RBCs from control fish, the levels of surface IgM were unchanged after six days of culture as determined by a two-way repeated measures ANOVA with Sidak’s multiple comparisons test. This stability is not characteristic of typical antigen-antibody complexes in live, non-fixed cells. n.s. P > 0.05; **P < 0.01; and ****P < 0.0001.

Given that only a fraction of IgM is specific to *T. bryosalmonae* and there is evidence of hypergammaglobulinemia (14) and polyreactivity in the Ig repertoire of fish with PKD (9), we hypothesized that some of the antibodies were bound to RBC antigens. Also, we previously observed feeding of a myxozoan on the RBCs of its fish host, incorporation of RBC antigens into/onto the parasite, and anemia (23). It is possible that there is also an exchange of parasite antigens onto the RBC surface. Since PKD is also caused by a myxozoan infection leading to anemia (24–26), it is possible that T. bryosalmonae incorporates its surface antigens onto the RBC surface. This may activate the classical complement pathway *via* IgM binding, lysing RBCs (27), causing IgM- and complement-dependent hemolytic anemia (28). To ensure that our hypothesis was plausible, we first tested for anemia and hypergammaglobulinemia. Indeed, diseased fish had 2.5 times fewer RBCs per µL on average (**Figure 3A**), and exhibited IgM hypergammaglobulinemia with approximately 8.5 times more serum IgM than control rainbow trout (**Figure 3B**).

To investigate whether the serum IgM recognizes RBC-specific, or *T. bryosalmonae*-derived antigens bound to the surfaces of RBCs, we incubated RBCs from either group (PKD or control) with pooled control plasma or plasma from infected fish. However, even after extended incubation, we were unable to induce the surface IgM phenotype (**Figure 3C**). The IgM mean fluorescence intensity (MFI) values did not change significantly in any of the tested groups, suggesting that the plasma of fish with PKD did not contain detectable IgM specific to either control RBCs or RBCs from fish with PKD. Consequently, we also did not detect hemolytic or cytolytic activity against RBCs (**Figure 3D**). The IgM from PKD plasma did not bind to an antigen on the surface of control RBCs and we did not observe hemolysis of these RBCs.

We ensured that there was no technical, procedural, or physiological limitations preventing activation of complement pathways by exposing rabbit whole blood to the same pooled fish plasma described above. Following the same experimental conditions, we observed a weak hemolytic activity in the samples exposed to 10% naïve fish plasma, 10% infected fish (PKD) plasma, or 50% naïve fish plasma. However, the treatment with the 50% plasma from infected fish eliminated approximately ~90% of rabbit cells (**Figure 3D**). Thus, despite the lack of binding to the fish RBCs, our results indicate that the hemolytic activity of sera is intact and substantially increased in the sera of fish infected with *T. bryosalmonae*. The observed lack of antibody- and/or complement-mediated lysis of trout RBC is therefore not due to inappropriate experimental conditions or unintended plasma inactivation.

Based on these results, it became increasingly unlikely that the IgM on the surface of RBCs was due to autoreactive antibodies bound to an RBC antigen. To support this hypothesis, we attempted to detect a loss of the surface IgM after *in vitro* culture of RBCs of PKD origin. We expected that both RBC surface antigens and the cognate antibodies would be internalized and turned over at a rate proportional to the half-life of the surface antigens. However, IgM on the surfaces of RBCs from PKD fish exhibited stability for at least six days after initial detection (**Figure 3E**). This is not typical for surface antigen interactions, with no significant change in the proportion nor MFI of mAb 1.14 staining (**Figure 3E**). Collectively, our results indicate that IgM on the RBCs of hosts with PKD is not due to antibody binding to an RBC self-antigen, whether one expressed in homeostasis or induced by disease. Similarly, due to the absence of hemolysis, our data do not support IgM being bound to a parasite antigen incorporated onto the RBC surface.

### 2.3 In infected fish, the RBCs acquire secretory IgM and express Mx proteins as well as MHC class I

**Figure 4:**
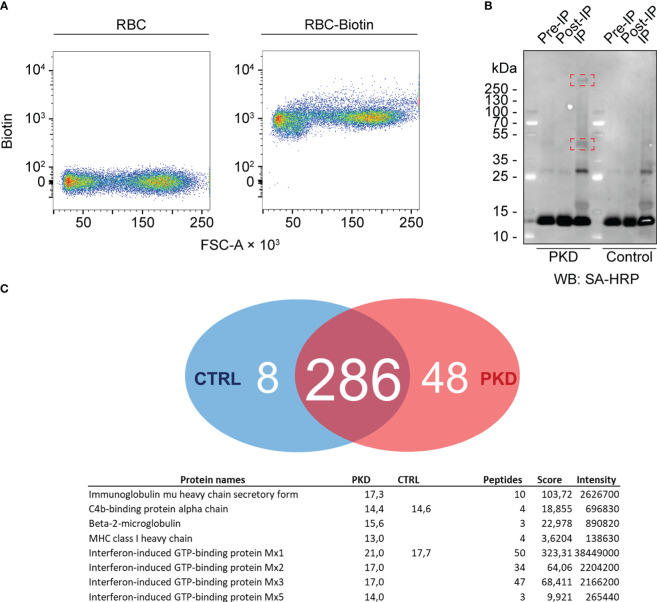
The cell surface proteome of red blood cells (RBCs)–differential surface proteins between RBCs from fish with proliferative kidney disorder (PKD) and control RBCs. **(A)** RBCs of fish with PKD or specific pathogen-free (control) fish were surface-biotinylated. Biotinylation was confirmed by comparing non-biotinylated RBCs and biotinylated cells (RBC-Biotin). The high affinity of biotin for avidin allowed us to detect biotin with streptavidin (a tetramer consisting of four molecules of avidin) conjugated to PE-Cy7. The PE-Cy7 tandem dye was detected in the infrared channel and its fluorescence measured by flow cytometry. Therefore, intensity of PE-Cy7 fluorescence is proportional to the levels of biotin and is represented on the y-axis (labeled “Biotin”) whereas the x-axis represents the forward scatter of these cells (FSC-A). **(B)** We determined any differences in cell surface proteins by lysing the cells. Again, we leveraged the affinity of avidin for biotin for immunoprecipitation (IP) of biotinylated proteins using ExtrAvidin-agarose resin, and western blotting (WB) with streptavidin-horse radish peroxidase (SA-HRP) (see “IP” lanes). Highlighted within red rectangles are one protein over 250 kDa and another approximately 48 kDa that are more abundant on the surfaces of RBCs from a fish with PKD. Comparing the signals from “Pre-IP” and “Post-IP” lanes faintly show depletion of these two proteins from the total lysate of IgM^+^ RBCs before IP (“Pre-IP”). **(C)** Several host proteins were differentially identified between PKD (48 exclusive proteins) and control (8 exclusive proteins) groups. Most prominently, the IgM heavy chain was identified exclusively from PKD RBCs. Surprisingly, an MHC class I orthologue and associated β2 microglobulin were also part of this group while results suggest that trout RBCs were responding to interferon during PKD.

To further elucidate the nature of IgM binding to the RBC surface, we investigated the changes in the surface proteome of RBCs by biotinylating RBC surface proteins from either infected or healthy fish using the membrane-impermeable EZ-Link Sulfo-NHS-LC-Biotin reagent (**Figure 4A**). We anticipated that overt differences would be readily distinguishable by western blot and further resolved by affinity purification and mass spectrometry. Western blotting of purified RBC from control and infected trout with mAb 1.14 revealed a high molecular weight band (over 250 kDa) that was present in infected fish (**Figure 4B**) but absent in the healthy control RBCs. This protein was detectable even without immunoprecipitation (IP) in the pre-IP whole cell lysate. Next, we immunoprecipitated the biotinylated proteins using avidin-agarose beads. These experiments revealed that, relative to an internal 13-kDa protein present at every step of the IP, an approximately 45-kDa molecule was enriched in fish with PKD indicating that there are proteomic differences between RBCs from infected and control fish.

Mass spectrometric analysis identified the IgM heavy chain exclusively from RBCs from fish with PKD (**Figure 4C**). Since our biotinylation strategy uses biotin that is membrane-impermeable, it labeled cell surface IgM which we immunoprecipitated and then detected by mass spectrometry. Importantly, the IgM identified on RBCs from infected animals was exclusively in secretory form. IgM exists as both a secreted multimeric form and a membrane-bound form. These forms are different enough to be distinguished by PCR as performed by Gorglione et al. (2013) (14) and by mass spectrometry here. The two forms have different C-termini for secretion or membrane anchoring (29). Here the peptides we identified mapped only to regions found on secretory IgM but not membrane-bound IgM, suggesting it is not directly anchored to the plasma membrane of RBCs.

Unexpectedly, interferon-stimulated gene products such as orthologues of Mx proteins and MHC class I (itself an interferon-stimulated molecule) with its associated light chain, β2 microglobulin, were also exclusive to the individual with PKD compared to control RBCs. Combined, these results suggest an activated profile and antiviral state in RBCs from rainbow trout with PKD.

Interestingly, we identified *T. bryosalmonae* antigens. Among those identified and mapped to the parasite’s transcriptome, many remain uncharacterized proteins with no known function. However, a few were identifiable exclusively in the PKD group and had homology to known proteins: unexpectedly, despite our biotinylation approach, the parasite peptides that could be identified were exclusively of intracellular origin and were housekeeping proteins such as a RhoA GTPase, a 40S ribosomal protein, tubulin, and an ADP ribosylation factor. These findings raise the questions of whether and how these parasite antigens are associated with the RBCs of the fish host.

### 2.4 RBCs from fish with PKD have altered metabolism, adhesion, and signaling

Assuming that the composition of fish plasma and the microenvironment surrounding RBCs were changed by *T. bryosalmonae* infection, we anticipated that surface IgM may be only one of many differences between RBCs from infected and control fish. To learn about the global changes that the RBCs have gone through, we turned to transcriptomics and analyzed the RBCs in bulk.

For this experiment, we selected Percoll-separated RBCs from four control fish and four infected fish. The four infected fish were at least 91.5% positive for mAb 1.14 staining as determined by the gating strategy shown in **Supplementary Figure 4**. To ensure the transcriptional changes of RBCs are not biased by an excess of contaminating leukocytes, we first confirmed that RBCs were Percoll-enriched and well separated from the leukocyte layer following density centrifugation. Preliminary experiments confirm that IgM is undetectable in the Percoll-separated RBC fraction (**Supplementary Figure 6A**). To this end, we performed analysis of expression of selected leukocyte lineage markers together with spectrin beta chain, encoded by *sptb*, that makes up part of the RBC cytoskeleton (**Supplementary Figure 6B**). In individual RBC specimens, our data indicate that *sptb* was over 180 times more abundant than either *cd8* or *cd4* T cell markers, the latter also serving as a monocyte/macrophage marker in teleost fish (30). Similarly, the relative levels of the B cell marker *igm*, and the thrombocyte marker *cd41* varied between about 10 times to over 10,000 times less than *sptb* in individual specimens. These data ensure that our enrichment protocols largely excluded non-RBCs from downstream analyses.

**Figure 5:**
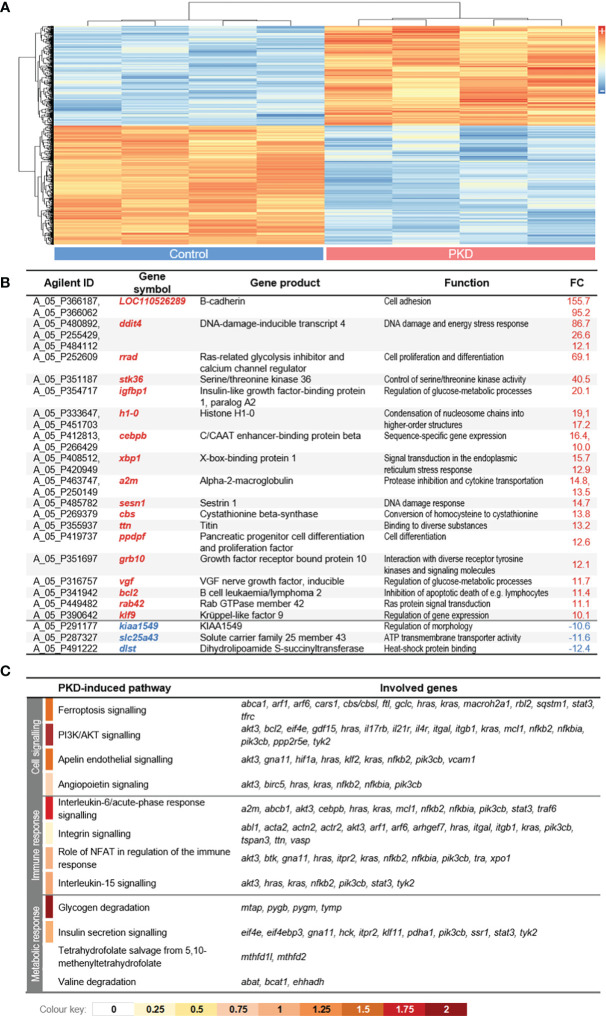
**(A)** Hierarchical clustering dendrogram of significantly differentially expressed (DE) genes (with |log2 FC|> 2; q < 0.01) from the comparison of control (left panel) versus red blood cells (RBCs) from fish with proliferative kidney disorder (PKD) (right panel). Samples from the infected group represent RBCs with between 91.5% to 95.3% positive mAb 1.14 staining as determined using gates and criteria that are shown in **Supplementary Figure 7** (examples of how we determined the percentage of mAb 1.14-positive cells). High and low expression intensities are represented by red and blue colors, respectively (see scale on the left margin). **(B)** List of DE genes with an absolute fold-change value >10 (excluding uncharacterized LOCs). Genes are listed as gene symbols along with the corresponding gene product and function. **(C)** List of the top four significantly enriched canonical pathways (with z ≥ 0) in control and PKD groups. The color code below the table displays the z-score values.

The complexity of plasma and its enhanced activity in fish with PKD (**Figure 3D**) led us to analyze its influence on the RBC transcriptome. Thus, we measured the transcriptomic changes in RBCs from infected rainbow trout compared to control rainbow trout by microarray analysis. In line with the proteomic data, the analysis provided clear evidence of a massively altered transcriptional profile in the RBCs of infected versus control fish. The transcript abundance of almost 750 features was modulated significantly (with *q* < 0.01) in RBCs from infected fish compared to the controls (**Figure 5A**), with 616 features representing non-duplicate, annotated genes. The proportion of up-regulated (n = 289; 46.9%) and down-regulated (n = 327; 53.1%) features in RBCs from diseased trout compared with controls was almost congruent. The most differentially expressed (DE) genes (over 10-fold change) are listed in **Figure 5B**. As already expected from the above findings, neither immunoglobulin-encoding nor associated transcripts were differentially expressed in either group. Gene ontology analyses of DE genes revealed the activation of various early pro-inflammatory pathways including *interleukin-6*, *acute-phase response signaling*, *integrin* and *NF-κB signaling* in RBCs from fish with PKD (**Figure 5C**). Concomitantly, later and more specific immune pathways were induced, including *role of NFAT in regulation of the immune response*, and *interleukin-15 signaling*. In the RBCs from infected fish, the pathways *PI3K/AKT signaling* and *apelin endothelial/angiopoietin signaling*, indicative of disease processes such as hemolytic anemia, were also activated (**Figure 5C**). Finally, the pathways controlling the *glycogen degradation* and *insulin secretion signaling* indicate increased erythropoiesis (31) and an increased energy demand of RBCs during PKD compared to physiological conditions. Altogether, regulation of the transcriptome and proteome of RBCs by PKD led to immune activation as well as metabolic reprogramming.

## 3 Discussion

We described a population of IgM^+^ RBCs in rainbow trout suffering from acute infection with *T. bryosalmonae*. In nearly all tested individuals with PKD, we detected surface IgM on the RBCs. The mass spectrometry analysis determined that the IgM was in secretory form and our experiments did not support binding of IgM to an antigen on control RBCs. Interestingly, the RBCs expressed innate immune signaling pathways that are conventionally associated with leukocytes.

Here, we place our observations in the context of what we already know about Igs and their roles in the immune system. Igs interact with other cells and molecules of the immune system *via* the Fab (fragment antigen-binding) and Fc (fragment crystallizable) regions—the antigen binding site and constant region, respectively. We and others observed IgM on RBCs (17). Studying the role of IgM on RBCs will teach us about the biology of nucleated RBCs and Ig function, as well as help us explore the potential of IgM as a marker of PKD.

### 3.1 Igs and their interactions with the host immune system

Conventionally, Igs are a product of B cells that encode the requisite and lineage-specific machinery such as recombination-activating genes (RAGs) for the somatic diversification and production of Igs. They exist as membrane-bound B cell surface receptors with a signaling component or as secreted soluble antibodies. Nonetheless, Igs are configured to interact with non-B cells that express compatible receptors.

In humans, the capture of free Ig is a feature limited to certain Ig isotypes, receptors, and leukocyte populations (32). Despite having identified both host and parasite proteins on the RBC surface (**Figure 4C** and data not shown), we cannot conclude whether the IgM we detected on RBCs binds to (parasite) antigens. Additionally, Abos et al.’s analysis of the antibody repertoire of PKD fish indicates that parasite-reactive antibodies were mostly of the IgT isotype (9). If they are not bound to the RBC surface as free IgM or bound to parasite antigens, it is possible that some of the IgM may be bound to self-antigens.

In complex with antigen, IgM can also exist in the form of complement-fixed immune complexes. Although complement is often associated with formation of the membrane attack complex, a less spectacular but more essential role of complement is in clearance of immune complexes. Senescent cells, the products of senescent cells, dying cells or their products (in the context of disease), and exogenous antigen (in contexts of infection) are turned over and cleared as complexes captured by RBCs (33). All the existing evidence suggests that fish express the necessary components for generation of complement-fixed immune complexes: complement fixation and antibody-dependent lysis of target cells have been established for decades in teleost fishes (34–36). Thus, bony fish RBCs may be able to capture immune complexes *via* complement receptors or independent of complement (16). Taking all possibilities into consideration will help explain this phenomenon we and others have observed in teleost fish.

### 3.2 IgM^+^ RBCs in a teleost fish

Chico et al. (17) also observed IgM^+^ RBCs in the same rainbow trout species (17). Comparing and contrasting our data suggests that they have different origins, one is heat stress-induced and the other related to a pathologically severe parasitic infection. The authors identified “shape-shifted” IgM^+^ rainbow trout RBCs that represent a morphologically distinct subpopulation as they demonstrated by flow cytometry and microscopy (17). We could not identify populations analogous to the “shape-shifted” IgM^+^ RBCs and we observed that over 95% of RBCs in an individual can be IgM^+^ (**Supplementary Figures 7, 8**). We observed an over one log-magnitude increase in MFI of mAb 1.14 staining in select fish RBCs (**Figure 1B**) which does not match the shift observed by Chico et al. (17). However, the two studies used a different anti-salmonid IgM monoclonal antibody (mAb 1.14 versus 1G7) (17). The authors also induced the phenotype *in vitro* whereas we could not, suggesting that the IgM on “shape-shifted” RBCs have an endogenous origin.

Irrespective of their origins, we acknowledge that many studies support that RBCs play an immunological role (16–19, 37–40)—in parasitic infection (this study), in the antiviral response, in general, and that this warrants further investigation.

### 3.3 RBCs maintain immune homeostasis in teleosts

Beyond interaction with IgM, our study along with others (16–19, 37–40) provide mounting evidence that nucleated RBCs interact with other components of the immune system and help maintain immune homeostasis. It is clear that RBCs have a larger role than delivering oxygen throughout the body and are more than bystanders during infection. Instead, in addition to immune complex turnover, nucleated RBCs express innate immune receptors, respond to stimulation, produce cytokines, and are phagocytic; even anucleate human RBCs were recently discovered to express cell surface TLR9 and detect CpG (41), suggesting that ancestral RBCs played a larger role in immunity.

Our study supports the findings that RBCs are responsive to immunological cues in their immediate microenvironment. We profiled a complex transcriptome suggesting that teleost RBCs express receptors for interleukins and interferons. These cells responded to cytokine cues by differential regulation of multiple interleukin signaling pathways that are rarely associated with RBCs. Given their pleiotropic effects, the induction of the PI3K/Akt and insulin signaling pathways may be indicative of not only metabolic reprogramming but also immunomodulation (42, 43). We also detected MHC class I on RBCs from fish suffering from PKD. This supports a report of this molecule being upregulated in brown trout following *T. bryosalmonae* infection (44) and a report of teleost RBCs encoding all the necessary machinery to act as antigen-presenting cells (16). Perhaps RBCs express the requisite integrins for homing to lymphoid tissues and priming of lymphocytes. Taken together, our findings suggest that teleost RBCs respond to and produce cytokine cues normally associated with leukocytes, and that they interact with the rest of the immune system.

Just how important could the RBC contribution be to immunity against pathogens, such as parasites in fish? In at least two models of myxozoan infection, the most severe forms of disease coincide with anemia. In *S. molnari* infection, the parasite directly interacts with RBCs and incorporates antigens from the RBC (23). If there is also an exchange of parasite antigens onto the RBC surface, the foreign antigens can potentially trigger antibody-mediated lysis of host RBCs or disrupt their function. However, we found no evidence of this strategy being used by *T. bryosalmonae*, with IgM and serum incapable of lysing host RBCs. Depending on the host and stage of the parasite, perhaps *T. bryosalmonae* instead encodes virulence factors that enable host manipulation and hemolysis (45). In future studies, we must determine if the parasite has a mechanism for depleting RBCs, and just how important anemia is for pathogenesis.

Type I interferon is associated with antiviral immunity. The type I interferon response as well as *mx* expression in rainbow trout RBCs are inhibited by viral haemorrhagic septicaemia virus (39). We also observed interferon responsiveness in RBCs and expression of Mx proteins albeit in parasite instead of virus exposure. In *T. bryosalmonae* infection/PKD, it is possible that parasite nucleic acids are transferred to RBCs for host manipulation. On the other hand, type II interferon responses can be protective against parasites as documented in a human cell line against the parasite *Toxoplasma gondii* (46). Given how multi-functional they are, by removing RBCs, is the capacity to detect, clear, and respond to the parasite compromised?

### 3.4 A model of PKD pathogenesis: An immune complex disease?

The progression of PKD is well documented: increasing parasite burden correlates with immunopathology in multiple organs, with a strong B cell/antibody component, and a cytokine expression profile that gives no clear indication of polarization towards any particular T helper cell subset (9, 14, 45, 47). Based on how the disease progresses, we propose a model of pathogenesis. In this model, the heavy parasite burden, systemic disease, and widespread immune activation produce an excess load of antigen. Together, kidney pathology and compromised erythropoiesis create the conditions for an excess of immune complexes whose clearance is disrupted by the depletion of the RBCs responsible for this task. In a vicious cycle, these immune complexes persist, are immunogenic *via* interaction with complement components and leukocytes, and perpetuate disease by causing further kidney damage and generating more immune complexes. Although we cannot support this model by comparing it to any documented disease in fishes, immune complexes can cause localized and systemic diseases in humans as exemplified by complement deficiency, systemic lupus erythematosus and immune complex nephritis (48–50). In contrast to humans and these chronic diseases however, the kidney of teleost fishes is hematopoietic and erythropoietic, making it plausible that the course and severity of disease are accelerated and exacerbated. We must test the plausibility of this model by studying the pathogenesis of PKD.

### 3.5 Implications for fish health and future directions

If the IgM we detected and the role of RBCs are central to the pathogenesis of PKD, then IgM may serve as a marker for diagnosis of PKD. We did not detect IgM on the surfaces of healthy RBCs. Also, the surface IgM levels of RBCs from infected fish declined in intensity between the 8^th^ and 9^th^ week of summer (**Supplementary Figure 7**). We hypothesize that the marker appears along with the disease, and that it diminishes during recovery from PKD. In our model, we speculate that IgM immune complexes accumulate following *T. bryosalmonae* infection of the kidneys, and deterioration of kidney function. Therefore, detecting these complexes on RBCs may correlate with infection and clinical signs of PKD, helping us diagnose the disease. In fact, secretory *igm* is already a correlate of parasite burden in PKD (14). Gene expression of *igm* positively correlates with expression of *T. bryosalmonae*-specific 60S ribosomal protein. It correlates with a complex immunological profile marked by *il10*, B cell markers of differentiation, and various regulators of T helper subset differentiation in the kidney (14). In other words, we may be able to measure IgM protein much like *igm* gene expression as a proxy for the immune response, severity of infection and the possibility of recovery. In practice and in contrast to gene expression profiling, the protein marker can be measured non-invasively using minute quantities of whole blood (as little as a μL).

Overall, these findings represent exciting opportunities to learn about basic teleost immunology. The mysteries surrounding the source and function of the surface IgM could be relevant to many topics in teleost immunology that we know little about: the non-redundant roles of different Ig isotypes; the interaction of antibodies with leukocytes; the turnover of immune complexes; the role of RBCs in immunity; comparative immunology. PKD may be a suitable model that helps solve some of these mysteries. Only then can we aspire to deriving diagnostic tests and therapeutic/prophylactic reagents like those readily available for mammalian diseases, but specific for fish diseases such as PKD.

## 4 Materials and methods

### 4.1 Experimental fish and diagnosis procedure

Animal procedures were performed in accordance with Czech legislation (section 29 of Act No.246/1992 Coll. on Protection of animals against cruelty, as amended by Act No. 77/2004 Coll.). Animal handling complied with the relevant European guidelines on animal welfare (Directive 2010/63/EU on the protection of animals used for scientific purposes) and the recommendations of the Federation of Laboratory Animal Science Associations. The animal experiments have been approved by the Ministry of Education, approval ID: MSMT-18301/2018-2.

Throughout the month of August 2020, we studied rainbow trout *O. mykiss* (mean body mass of 120 g) reared in a commercial inland fish farm, in the South Bohemian region of the Czech Republic. Fish displaying visible signs of PKD such as pale gills, abdominal distension, and lethargy were transported alive in oxygenated water/bags to the Institute of Parasitology of the Biology Centre of the Czech Academy of Sciences (České Budějovice, Czech Republic). These fish were compared to an additional 15 naïve SPF fish (mean body mass of 30 g), reared in experimental recirculating systems of the Faculty of Fisheries and Protection of Waters, University of South Bohemia in the Czech Republic. Data from these fish are referred to as coming from “control” fish in the text.

Upon arrival, we euthanized fish with clove oil and bled them from caudal veins using heparinized syringes and needles. Upon dissection, we prepared imprints from their swollen kidneys and stained them according to the manufacturer’s instructions with the Kwik–Diff Kit (Richard Allen Scientific, San Diego, CA, USA), consisting of methanol fixation followed by eosin and methylene blue staining. Light microscopy was used to identify *T. bryosalmonae* parasites. All fish in the infected cohort had kidney swelling indices between 3-4 according to the scale and parameters established by Clifton-Hadley et al. (20) and variable signs of spleen swelling, fibrosis, and of gill and liver pallor.

### 4.2 Initial magnetic-activated cell sorting enrichment of IgM^+^ RBCs

We initially identified the population of IgM^+^ RBCs serendipitously when studying the B lymphocyte population in trout lymphoid organs (**Supplementary Figure 3**). Splenic and anterior kidney tissues were initially passed through 100-μm cell strainers (Corning, Durham, NC, USA) using a combination of dissociating the tissue with the textured end of a syringe plunger and washing with Iscove’s Modified Dulbecco’s Medium (IMDM, Life Technologies Limited, Paisley, UK). This process began with placing the tissue onto the mesh of the cell strainer. We added 1 mL of IMDM onto the tissue to prevent it from drying. We repeatedly applied gentle force and a twisting motion with the syringe plunger. We then washed the dissociated tissue. Each wash consisted of pipetting 1 mL of IMDM onto the strainer, focusing the wash on areas of the strainer with tissue. Three washes were performed in total. This is how we excluded cellular aggregates larger than 100 μm. For this purpose, the IMDM was not supplemented or added with any fetal bovine serum or antibiotics. We then loaded the cell suspension onto 25% Percoll (one part Percoll diluted in three parts IMDM, no other component was added) (Percoll purchased from Cytiva, Uppsala, Sweden) for centrifugation at 500 *g* for 15 min (gentle acceleration and braking) to exclude cells of lower density such as adipocytes and fibroblasts. After we washed away the remaining Percoll with excess IMDM (no additives), we counted 10 million cells as input material for the MACS. They were stained with 100 ng of mAb 1.14 specific to salmonid IgM (21, 22), obtained from Dr. Bernd Köllner (Institute of Immunology of the Friedrich-Loeffler-Institute, Federal Research Institute for Animal Health). The IgM^+^ cells were positively selected using MACS and anti-mouse IgG microbeads and LS columns (both purchased from Miltenyi Biotec, Bergisch Gladbach, Germany) according to the manufacturer’s instructions, with purity assessed by flow cytometry on a BD FACSCanto II (BD Biosciences, Prague, Czech Republic). All flow cytometry data here and throughout the manuscript were analyzed with the FlowJo v10 software (Becton, Dickinson and Co., Ashland, OR, USA).

### 4.3 Trout RBC isolation, storage, and culture

The heparinized whole blood of trout was either tested whole without separation, or RBCs were separated from the whole blood of PKD and control rainbow trout. For separation, it was achieved by diluting blood 1:1 with IMDM (no serum nor antibiotics added) before loading onto 51% Percoll consisting of 5.1 parts Percoll (GE Healthcare, Uppsala, Sweden) combined with one part 10× PBS (Life Technologies Corporation, Grand Island, NY, USA) and 3.9 parts distilled water, followed by density centrifugation at 500 *g* for 8 min with gentle acceleration and braking. We then washed cell pellets twice with IMDM.

In preliminary experiments, the 51% Percoll enriched for RBCs, and depleted lymphocytes and their endogenous products or surface antigens (such as IgM) from downstream analyses (**Supplementary Figure 6**). These cells were lysed at -20 °C and later thawed for downstream western blotting. A portion of cells was cultured in IMDM, 10% fetal bovine serum (Biosera Europe, Nuaillé, France), and further supplemented with a solution of antibiotic-antimycotic (Life Technologies Limited, Paisley, UK) and GlutaMAX (Life Technologies Limited, Paisley, UK), both diluted to 1×. Another portion was washed three times with PBS, then aliquots of 25 × 10^6^ cells were pelleted, and either stored in RNAlater (Sigma-Aldrich, Saint Louis, MO, USA), or biotinylated (see Materials and Methods, section 4.8).

### 4.4 Flow cytometry detection of surface IgM

For the staining of whole blood, 2 μL of the specimen was washed twice with IMDM (all washes and stainings were in the absence of antibiotics or bovine serum) in order to remove trout serum IgM. We stained the cells with 100 ng of mAb 1.14 diluted in IMDM for 20 min in ice. We washed away excess mAb 1.14 with IMDM before incubating cells with either 1:500 goat anti-mouse IgG-Alexa Fluor 488 (Invitrogen, Rockford, IL, USA) or 1:500 goat anti-mouse IgG1-APC (Abcam, Cambridge, UK), both diluted in IMDM. We then incubated the cells for 20 min in ice before washing away excess antibody. Staining with the goat anti-mouse IgG-Alexa Fluor 488 was verified by flow cytometry before we proceeded with the protocol in Materials and Methods 4.5 for fluorescence microscopy. To the cells stained with the goat anti-mouse IgG1-APC, we added propidium iodide (0.4 μg/mL) (Life Technologies, Eugene, OR, USA) (representative staining presented in **Supplementary Figure 8**) and measured the cells on the BD FACSCanto II.

In some instances, to ensure we could distinguish leukocytes from RBCs during flow cytometry analysis, we also further incubated cells in 5 μg/mL 3,3’-dihexyloxacarbocyanine iodide [DiOC6 (3)] (Life Technologies, Carlsbad, CA, USA) diluted in IMDM. See **Supplementary Figure 4** for the gating strategy. For Percoll-separated RBCs, we followed the same procedure but initiated the protocol with 2 million RBCs rather than 2 μL of whole blood.

We used a two-laser configuration of the BD FACSCanto II: equipped with a 488-nm blue laser and a 633-nm red laser. We detected the fluorochromes through the green channel (for Alexa Fluor 488 or DiOC6), the red channel after excitation with the 633-nm laser (for APC) or another red channel following excitation with the 488-nm laser (for propidium iodide)

We did not fix or permeabilize cells for any experiment described here. Therefore, we detect only IgM on the surfaces of RBCs.

All data summarizing flow cytometry results were prepared, analyzed, and presented *via* Prism 9 (GraphPad Software, Boston, MA, USA).

### 4.5 Fluorescence microscopy detection of surface IgM

A portion of cells fully stained with mAb 1.14 and anti-mouse IgG-Alexa Fluor 488 were allocated for microscopy by centrifugation followed by a 20 min incubation at ambient temperature with 200 μL of the cell-permeable NucBlue Live ReadyProbes Reagent (Life Technologies, Carlsbad, CA, USA), prepared according to the manufacturer’s instructions. We prepared slides by cytospinning these cells in 60 mm^2^ cyto chambers (Hettich, Tuttlingen, Germany) and centrifuging at 500 *g* for 5 min before analyzing them on an Olympus BX51 (Olympus, Japan). These reagents were detected by UV excitation and the filter set in Position 4 for the NucBlue. The Alexa Fluor 488 signal was detected by blue excitation and the filter set in Position 2.

### 4.6 Western blot detection of rainbow trout IgM

From the heparinized blood of fish, we centrifuged the blood in order to collect plasma or cells for downstream analyses. Plasma (diluted to 3.75% with PBS and Laemmli sample buffer [Bio-Rad, USA]) was loaded into the wells of 12% polyacrylamide gels. After SDS-PAGE, we transferred gel contents onto TransBlot Turbo mini-size PVDF membranes (Bio-Rad, USA) and blocked them for 30 min at room temperature with constant shaking using 7% blotting grade skimmed milk powder (mass over volume, g/mL) (Carl Roth, Karlsruhe, Germany) diluted in TBS 0.1% Tween 20 (TBST, one part of Tween 20 added to 1000 parts of TBS). The membranes were incubated overnight at 4°C with mAb 1.14 diluted to 1 μg/mL in 5% skimmed milk in TBST. We washed the membranes three times for 5 min each in TBST before incubating them with anti-mouse IgG conjugated to horseradish peroxidase for one hour in 5% skimmed milk in TBST. Finally, we washed the membranes thrice for 5 min per wash and exposed membranes to Clarity Western ECL Substrate solution (Bio-Rad, USA). The chemiluminescent signal was photographed on a ChemiDoc MP Gel Imaging System (Bio-Rad, USA) using the “optimal exposure” setting.

Alternatively, we also detected IgM from the WBC fraction of Percoll-separated blood (**Supplementary Figure 6A**) by western blotting. These samples come from cells enriched by 51% Percoll separation. They were centrifuged at 500 *g*, washed three times with IMDM (without serum and without antibiotics), before they were frozen at -20°C for storage and later thawed for western blotting according to the protocol above.

Densitometry was performed using ImageJ version 1.53 (National Institutes of Health, Bethesda, MD, USA) and the data was summarized and analyzed in Prism 9.

### 4.7 Hemolysis of target trout or rabbit blood cells

Either fresh or cultured trout cells (prepared as detailed above), were incubated with 50 µL of 50% or 10% plasma, pooled from equal volumes of plasma from either five infected or five control fish, and diluted in IMDM with no fetal bovine serum nor antibiotics. The purified RBCs from either PKD or healthy animals were incubated with the fish plasma at ambient temperature (approximately 20°C) for 30 min before we added an equal volume of propidium iodide to a final concentration of 0.4 µg/mL.

Coagulation of rabbit whole blood was inhibited with heparin. A million rabbit blood cells were centrifuged at 250 *g* and incubated with 50 µL of the same pooled plasma described above. After a 30-min incubation at ambient temperature, 50 µL of propidium iodide was added to a final concentration of 0.4 µg/mL.

The number of surviving propidium iodide-negative cells was measured. Additionally, with a set and constant duration of acquisition and a medium flow rate (60 µL/min) on the FACSCanto II, we were able to determine the proportion of surviving cells out of the initial cells plated for the reaction (**Supplementary Figure 8**).

### 4.8 Biotinylation and mass spectrometry identification of RBC surface proteins

To label the surface proteome of IgM^+^ RBCs, we biotinylated 25 × 10^6^ live Percoll-separated cells using EZ-Link Sulfo-NHS-LC-Biotin (Pierce Biotechnology, Rockford, IL, USA) according to the manufacturer’s instructions and by incubating cells with the reagent for 30 min at 4 °C. Following PBS glycine washes, we performed two additional PBS washes.

Following this procedure, cell viability was confirmed by light microscopy and successful biotinylation was confirmed by flow cytometry using streptavidin-PE-Cy7 (Life Technologies, Carlsbad, CA, USA) at 1:500 in PBS. This fluorochrome was detected in the infrared channel following excitation with the 488-nm blue laser.

Next, we stored cells frozen until we were ready to use them, we then thawed cells, supplemented the lysate with cOmplete, EDTA-free protease inhibitor cocktail (Roche Diagnostics, Mannheim, Germany) and the lysate was collected after centrifugation at 12,000 *g* for 10 min at 4 °C, leaving behind the pellet and membrane fraction. We proceeded by lysing 20 × 10^6^ cells for IP (via freezing at 20 °C for at least one day before thawing on ice) using ExtrAvidin-Agarose (Sigma-Aldrich, Saint Louis, MO, USA). The beads were first washed three times in PBS. The lysate was topped up to 700 μL with protease inhibitor-supplemented PBS (the 700 μL ensures that the beads will not dry during the IP). 15 μL of this solution was aliquoted and denatured with 5 μL of 4× NuPAGE LDS Sample Buffer (Invitrogen, Carlsbad, CA, USA) as a “pre-IP” specimen. 20 μL packed bead volume of washed ExtrAvidin-Agarose was then added to the lysate and the solution was left to rotate overnight at 4 °C. We centrifuged the beads for 1 min at 200 *g*, let the beads settle for 1 min before 15 μL of lysate was aliquoted and denatured as above but this time, the lysate represents a “post-IP” specimen. We washed the beads five times after which 5% of the beads were set aside to verify that the IP was successful. Using this 5% of beads, the biotinylation was confirmed and the biotinylated proteins detected by western blot using 1:5000 streptavidin-horseradish peroxidase (Thermo Fisher Scientific, Rockford, IL, USA).

### 4.9 NanoLC ESI-MS/MS protein identification

Proteins were identified after tryptic digestion by mass spectrometry. Microbeads with immobilized proteins were washed in 100 mM ammonium bicarbonate. Reduction and alkylation were performed using 10 mM dithiothreitol (45 min at 56°C) and 55 mM idoacetamide, respectively. Alkylation was stopped after 20 min by addition of 55 mM dithiothreitol. Protein digestion was performed directly on the microbeads by addition of 0.15 µg of trypsin and overnight incubation at 37°C. The digestion was terminated by addition of formic acid to a final concentration of 2.5%. The obtained peptide mixtures were purified using C18 Empore™ disks (3M, USA) (51). Peptides were dissolved in 20 µL of 3% acetonitrile/0.1% formic acid. The analysis was carried out on an UltiMate 3000 RLSCnano system (Thermo Fisher Scientific, USA) coupled on-line to mass spectrometer timsTOF Pro (Bruker Daltonics, Germany). The peptide solution was injected onto an Acclaim™ PepMap™ 100 C18 trapping column (300 µm i.d., 5 mm length, particle size 5 µm, pore size 100 Å; Thermo Fisher Scientific, USA) using a 2 µL injection volume and a 2.5 µL/min flow rate for 2 min. Bond peptides were eluted from a trapping column onto an Acclaim™ PepMap™ 100 C18 trapping column (75 µm i.d., 150 mm length, particle size 2 µm, pore size 100 Å; Thermo Fisher Scientific, USA) and separated by a 48 min-long linear gradient of 5-35% acetonitrile/0.1% formic acid at a constant rate of 0.3 µL/min. The column oven temperature was set at 35°C. The MS measurement was operated in PASEF scan mode with positive polarity. Electrospray ionization was performed using a CaptiveSpray (Bruker Daltonics, Germany) with capillary voltage at 1500 V, dry gas at 3 L/min and dry temperature at 180°C. Ions were accumulated for 100 ms, and 10 PASEF MS/MS scans were acquired per topN acquisition cycle. An ion mobility range (1/K0) was set at 0.6-1.6 Vs/cm^2^. Mass spectra were collected over a *m/z* range of 100 to 1700. The polygon filtering was applied to exclude the low *m/z* of singly charged ions. The target intensity was set at 20,000 to repeatedly select precursor for PASEF MS/MS repetitions. The precursors that reached the target intensity were than excluded for 0.4 min. Collision energies were changed from 20 to 59 eV in 5 steps of equal width between 0.6 and 1.6 Vs/cm^2^ of 1/K0 values.

Proteins were identified by raw MS data processing in MaxQuant software (version 1.6.14) (52) with integrated Andromeda search engine (53). Oncorhynchus mykiss database downloaded from Uniprot (16. 09. 2020) supplemented with protein sequences from PKD transcriptomes (45) and contaminant database included in MaxQant software were used to search proteins. The default parameters for TIMS-DDA search type and Bruker TIMS instrument were applied. Trypsin/P was set as enzyme allowing up to two missed cleavages in specific digestion mode; carbamidomethylation of cysteine was used as fixed modification; methionine oxidation and protein N-term acetylation were set as variable modifications; the minimum required peptide length was set to seven amino acids. Precursor ion tolerance was set at 20 and 10 ppm in first and main peptide search, respectively; the mass tolerance for MS/MS fragment ions was set at 40 ppm; peptide and protein identifications were filtered using a target-decoy approach at a false discovery rate (FDR) of 1%. Label-free quantification (LFQ) of proteins was done using the algorithm integrated into MaxQuant software with minimum ratio count set at 2. LFQ intensity values were log2-transformed. Proteins identified by only 1 peptide were further excluded from data set.

### 4.10 RNA preparation

The Isolate II RNA Micro Kit (Bioline/Meridian Bioscience, Memphis, TN, USA) was used to extract and purify total RNA from RBCs. The concentration and purity of the isolated RNA were determined using the NanoDrop One spectrophotometer (NanoDrop Technologies/Thermo Fisher Scientific, USA). The integrity of RNA was evaluated using the Agilent 2100 Bioanalyzer (Agilent Technologies, Santa Clara, CA, USA) and the Eukaryote Total RNA Nano Assay (Agilent Technologies, Santa Clara, CA, USA) revealing RIN values ranging from 8.3 to 10.

### 4.11 Array hybridization and analysis

We isolated individual RNA specimens from the RBCs of four healthy (C1-C4) and four infected rainbow trout (PKD1-PKD4). The RBCs from fish with PKD ranged between 91.5% and 95.3% positive for mAb 1.14 staining (see **Supplementary Figure 7** for example gating, criteria, and how we determined the percentage of mAb 1.14-stained cells). The presence of leukocyte and thrombocyte markers was also evaluated by real-time quantitative PCR (RT-qPCR) using the LightCycler 96 System (Roche, Germany) in combination with the SensiFAST SYBR No-ROX Kit (Bioline, London, UK). We profiled the marker genes encoding *sptb* in RBCs (5’-CACGCATCAAAACCCTAACAGAT-3’, sense; 5’-TGATGAGCATGCGTCCATCCC-3’, antisense), *cd8* on cytotoxic T cells (5’-TTTTGTCAAGAAACTCTCCAACTGA-3’, sense; 5’-AATCACCATGTTGCTCTTAGTCTT-3’, antisense), *cd4* on T-helper cells (5’-ATCTCCTCAACAGGGGCTGAAG-3’, sense; 5’-CTGTCTTTCCACTCTGGATCTAT-3’, antisense), *igm* on B cells (54), and *cd41* on thrombocytes (54). *Eef1a1* and *rps5* served as reference genes to normalize the expression data. The results of this analysis are presented in **Supplementary Figure 6**.

Then, the RNA specimens were converted to Cy3-labeled cRNA and hybridised with 8×60 K Agilent Salmon Oligo Microarrays (ID 020938, Agilent Technologies, Santa Clara, CA, USA; GEO platform: GPL21057) following the Agilent 60-mer oligo microarray processing protocol.

The fluorescence signals of the hybridised Agilent microarrays were scanned with a G2505C Microarray Scanner System (Agilent Technologies, Santa Clara, CA, USA) at a resolution of 2 µm. The Agilent Feature Extraction Software (FES) 10.7.3.1 was used to read and process the microarray-image files with standard settings. The FES corrected the background using a two-sided Student *t*-test. The features that passed this quality control were further analysed with the limma package of R (version 3.6.3) (55). Following quantile normalisation, pairwise comparisons of the transcript abundances from samples from PKD versus control individuals were employed. To control the false discovery rate, *p*-values were adjusted (56) and only genes with an adjusted *p*-value (*q*-value) of < 0.01, and an absolute fold change of > 2.0 were considered DE genes and included in further data processing. The reliability of the microarray-predicted data has previously been proven in various qPCR studies from our group (56–60). The full complement of microarray data were deposited in the NCBI database Gene Expression Omnibus (GEO, http://www.ncbi.nlm.nih.gov/geo/; accession: GSE198859).

DE genes were re-annotated using the Basic Local Alignment Search Tool (BLAST) considering only transcripts with unique BLAST results (coverage and sequence identity of > 80%). Only 6.95% of all DE genes could not be annotated. Hierarchical/K-means clustering and heat-map matrix analyses were performed using the R packages heatmap.2, factoextra and cluster. A functional analysis was performed using the Ingenuity program (Ingenuity Pathway Analyses/Qiagen) to evaluate canonical pathways, which are indicated in the following sections by italic face. Benjamini-Hochberg multiple-testing was performed and *p*-values > 0.05 were considered as a cut-off score. The output lists were carefully reviewed and all those pathways and functions were deleted, which either are associated with a mammalian disease or occur in a specific cell type other than the analysed one. All relevant pathways are indicated in the following sections by italic face. The z-scoring system was used to evaluate whether a particular pathway was activated (z > 1) or repressed (z < 1).

## Data availability statement

The datasets presented in this study can be found in online repositories. The names of the repository/repositories and accession number(s) can be found below: https://www.ncbi.nlm.nih.gov/geo/, GSE198859.

## Ethics statement

The animal study was reviewed and approved by the Ministry of Education, Youth and Sports.

## Author contributions

JC, AP-S, JM, and TK conceptualized the idea for the article, designed the methodologies to answer the research questions, performed experiments, analyzed the data, and wrote the original draft of the article. AR and DK performed the microarray analyses and formally analyzed the transcriptomics data. FD performed the mass spectrometry and analyzed the data. TK acquired funding and supervised the research. AH acquired funding and supervised the project. All authors contributed to the article and approved the submitted version.
