# Supplementary material for: Red blood cells in proliferative kidney disease—rainbow trout (Oncorhynchus mykiss) infected by Tetracapsuloides bryosalmonae harbor IgM+ red blood cells
Source: Front Immunol. 2023 Feb 15;14:1041325. doi: 10.3389/fimmu.2023.1041325 (PMC9975563; doi:10.3389/fimmu.2023.1041325)
Supplement: Supplementary file 1 [file DataSheet_1.pdf]

Supplementary Material

Supplementary Figures

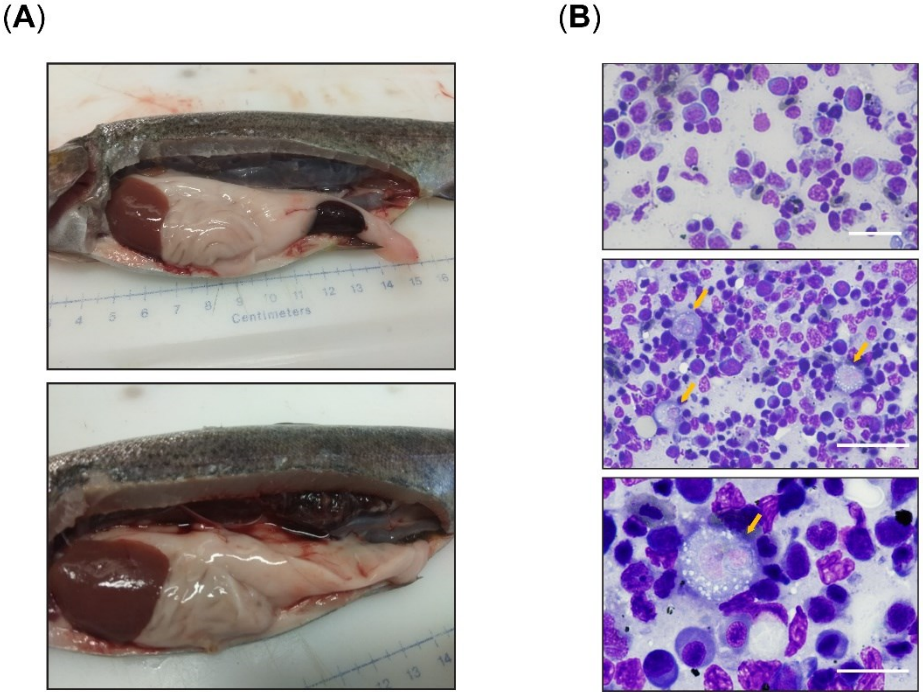

Supplementary Figure 1 | Kidney hyperplasia, a clinical sign of proliferative kidney disease, as well as extrasporogonic-stage *T. bryosalmonae* were observed in rainbow trout. **(A)** We dissected several commercially farmed rainbow trout with signs of PKD such as abdominal distension. The kidneys of these fish were swollen which is an additional sign of PKD. **(B)** Kwik-Diff smears of the anterior kidney of these fish (middle and bottom images) revealed the presence of extrasporogonic-stage *T. bryosalmonae* (orange arrows) whereas we were not able to identify the parasite in healthy fish (topmost image). The scale bar in the top two images represent 50 µm whereas the lower one represents 20 µm.

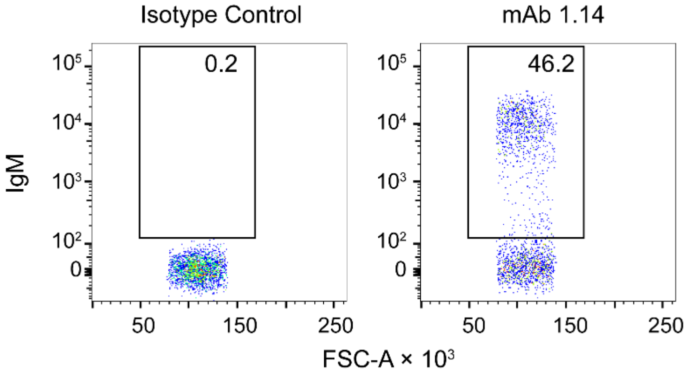

Supplementary Figure 2 | IgG1 isotype control flow cytometry stain of 51% Percoll-separated control rainbow trout whole blood (left) versus staining of the same specimen using mAb1.14 (right). As an isotype control, we used anti-carp IgM (clone WCI12) that is a mouse monoclonal antibody of IgG1 isotype like mAb 1.14. Numbers in boxes/gates indicate the percentage of detectable IgM^+ blood cells among lymphocytes.

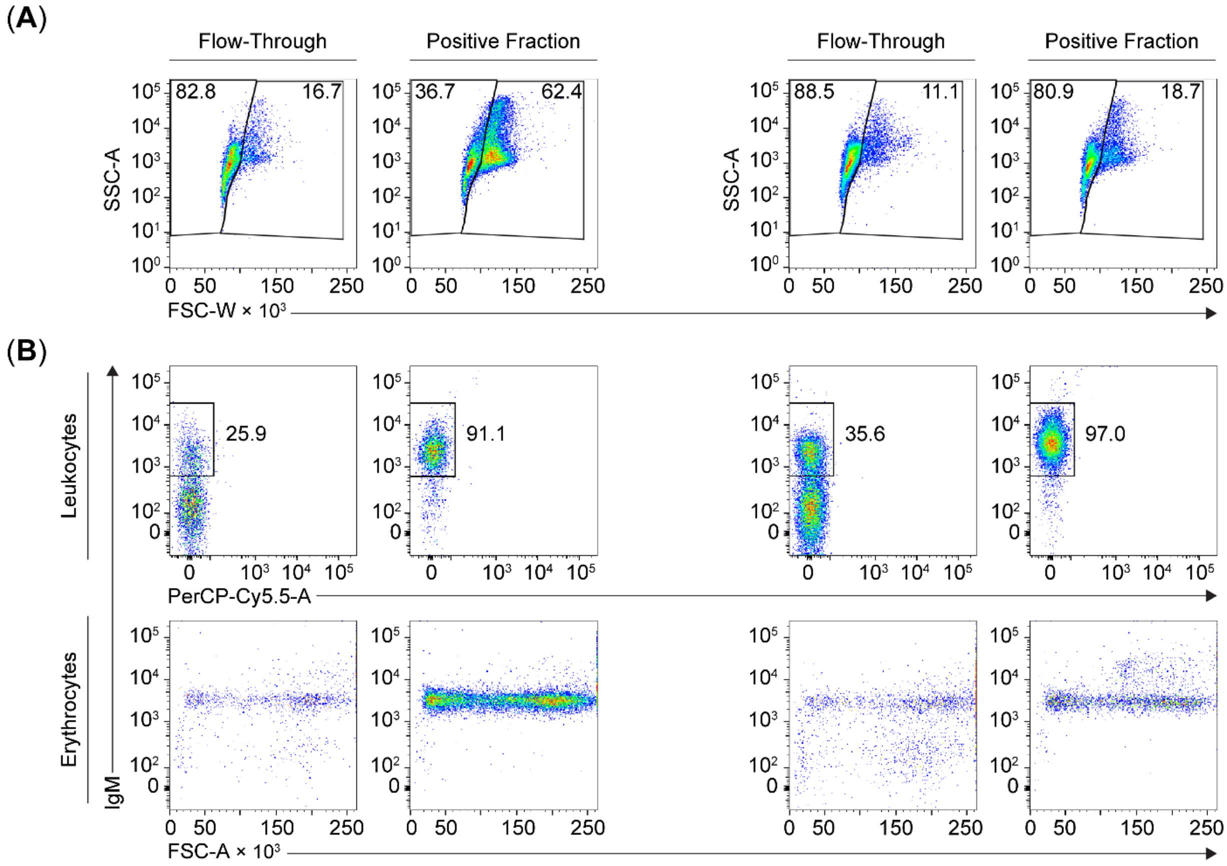

Supplementary Figure 3 | In a rainbow trout with PKD, MACS IgM positive selection enriches not only lymphocytes but also IgM$^+$ red blood cells (RBCs) from the spleen and anterior kidney. **(A)** This row of plots represents total cells from either the spleen (first two plots on the left) or the anterior kidney (next two plots on the right). Each compartment is labeled at the top of the figure. Within each compartment we present the result of the IgM positive selection with cells from the column flow-through or the positive fraction (please, refer to labels). Gates within each plot help to distinguish leukocytes from RBCs/erythrocytes based on side scatter area (SSC-A) and forward scatter width (FSC-W). E.g., in the spleen, RBCs represented 16.7% and 62.4% of all cells in the MACS flow-through and positive fraction, respectively; leukocytes represented 82.8% and 36.7% of all cells in the MACS positive fraction, respectively. **(B)** Following the same organization of compartment and MACS fractions as in **(A)**, this section presents the levels of IgM (y-axis) on the surfaces of leukocytes (upper row) or RBCs (lower row). Parameters on the x-axes were chosen to help gating on IgM$^+$ leukocytes (PerCP-Cy5.5-A, top row) or to help confirm the identity of RBCs which are very diverse in FSC-area (FSC-A, bottom row). Regardless of the compartment, IgM$^+$ cells are enriched to over 90% purity among leukocytes. Surprisingly, RBCs were also enriched in the positive fraction and have detectable surface IgM relative to the majority of leukocytes in the flow-through. Although these RBCs may be contaminants from the dissection, they led us to investigate and confirm this phenotype in the blood and in other individuals with PKD.

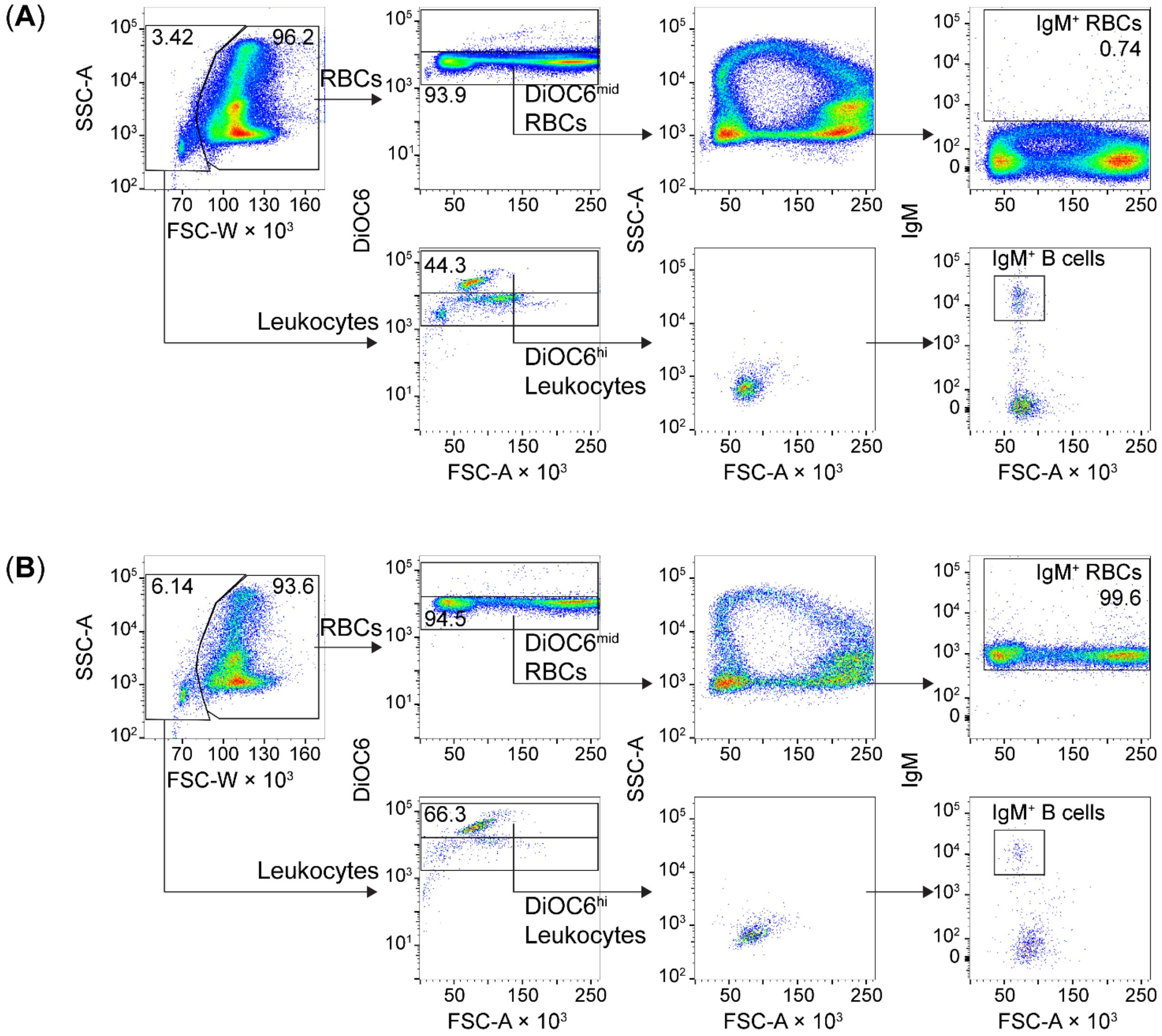

Supplementary Figure 4 | Gating strategy for distinguishing leukocytes from red blood cells (RBCs) from either **(A)** whole blood from control fish or **(B)** whole blood from infected fish. The legend and description that follow are identical for both **(A)** and **(B)**. Arrows indicate which subpopulation we select and guides the reader to the next plot representing cells from that subpopulation. From left to right, we sequentially gate on subpopulations of RBCs (top row) or leukocytes (bottom row) to analyze their scatter profile or levels of surface IgM. (Top left panel) RBCs can be largely identified by their distinct side scatter area (SSC-A) versus forward scatter width (FSC-W) profile. This strategy alone is not perfect but can be improved with the endoplasmic reticulum-specific reagent DiOC6 (second column). The dye distinguishes the more active leukocytes by their higher endoplasmic reticulum content and therefore higher DiOC6 staining intensity. In the second column, we gate on the DiOC6-high population which represent leukocytes (DiOC6^{hi}) and the DiOC6-mid population which have a weaker staining intensity and represent the RBCs (DiOC6^{mid}). In the third column of plots, we analyze the SSC-A versus FSC-A profile of the RBCs (top) or leukocytes (bottom). These parameters reflect cell density/granularity and cell size, respectively. The final row of plots presents the levels of IgM for the RBCs (top) or leukocytes (bottom). Only in **(B)** can we detect a significant portion of IgM$^+$ RBCs.

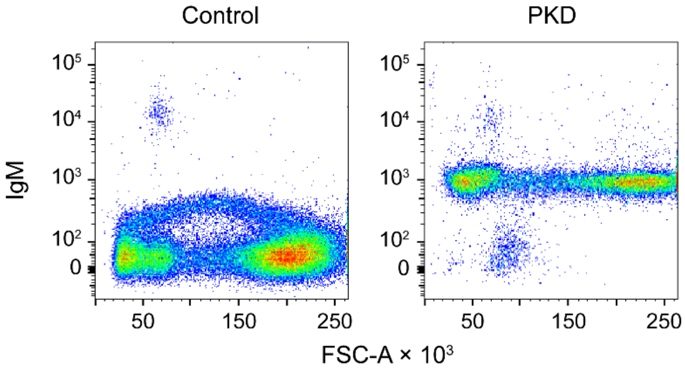

Supplementary Figure 5 | Flow cytometry profile of whole blood cells from either a control fish (left) or a fish with proliferative kidney disease (PKD) (right). Plots represent total non-gated blood cells from these fish and present their surface IgM versus forward scatter area (FSC-A) profiles. The results are analogous to those presented in **Figure 1A** and **2C**. Due to internal positive and negative controls (the IgM^+ and IgM^- leukocytes, respectively), both flow cytometry and microscopy independently confirm that the IgM^+ RBCs are not a product of a technical error. E.g., increased instrument signal amplification/voltage in the PKD specimen relative to the control (flow cytometry) or overexposure of the PKD specimen relative to the control in microscopy.

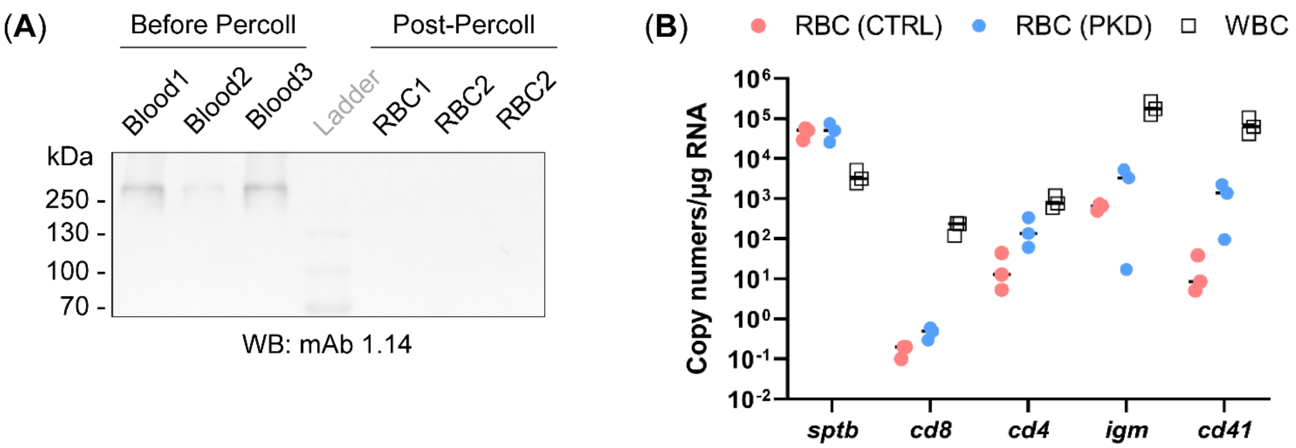

Supplementary Figure 6 | Percoll density centrifugation excludes lymphocytes from downstream analyses. **(A)** We separated rainbow trout whole blood by 51% Percoll density centrifugation and sought to detect IgM in blood specimens before separation and in the red blood cell (RBC) pellet after separation. We present a mAb 1.14 western blot of representative specimens from individual specific pathogen-free carp demonstrating absence of IgM and purity in the RBC fraction. The lysate of approximately 10^6 cells were loaded into each well. The high molecular weight band matches the one seen in plasma (**Figure 3B**). **(B)** In parallel, using real-time quantitative PCR (RT-qPCR) we verified that Percoll density centrifugation enriched the RBC population for downstream RBC-specific proteomic and transcriptomic analyses. Following 51% Percoll density centrifugation, we collected the cell pellets and measured the expression of the following markers, each associated with a particular blood cell population (in brackets): *sptb* (RBCs), *cd8* ($CD8^+$ T cells), *cd4* ($CD4^+$ T cells), *igm* (IgM^+ B cells), and *cd41* (thrombocytes). The filled red circles, filled blue circles, and open squares represent respectively density centrifugation-enriched control RBCs, RBCs from fish with PKD, or the white blood cells (WBCs) in the buffy layer from individual fish.

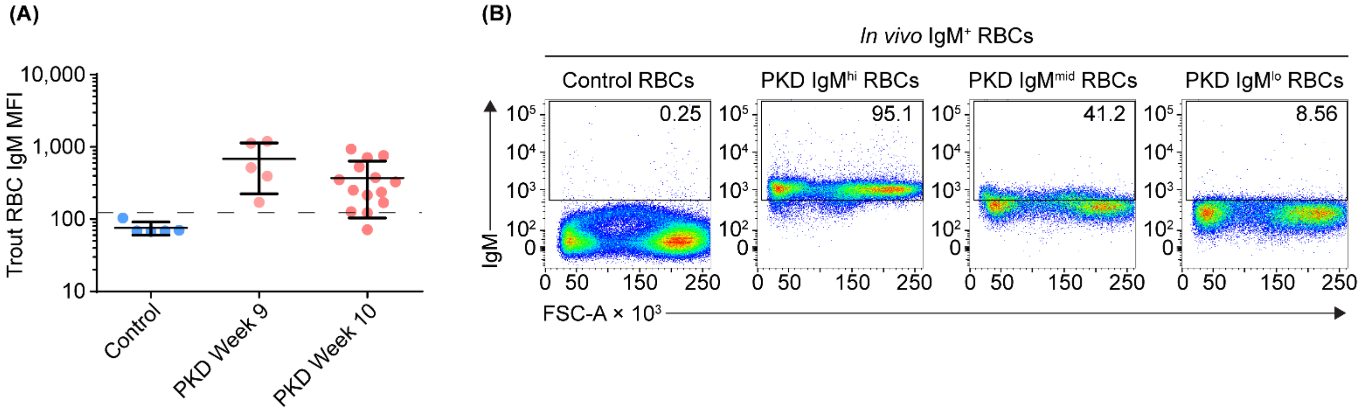

Supplementary Figure 7 | **(A)** We parsed data previously presented in **Figure 1B** into the weeks in which they were collected. We measured the level of IgM on the red blood cells (RBCs) during the eighth and ninth weeks of summer. Between these timepoints, we observed a downward trend in amount of IgM detected such that by week 9, the intensity of mAb 1.14 staining of RBCs from one fish fell below the threshold of the mean of the control group plus three SDs (dashed horizontal grey line), whereas the RBCs from two other fish were borderline above the threshold. **(B)** Here, we illustrate the flow cytometry profile (forward scatter-area [FSC-A] versus intensity of IgM staining) of the RBCs from various fish. Shown are three representatives of the different levels of surface IgM we measured in individual fish with PKD, ranging from high mAb 1.14 staining (IgM^{hi}) to low mAb 1.14 staining (IgM^{lo}). For important experiments such as the microarray analysis, the four RBC samples selected were all from the category IgM^{hi} shown here (Results section 2.4; Materials and Methods, section 4.11; **Figure 5**).

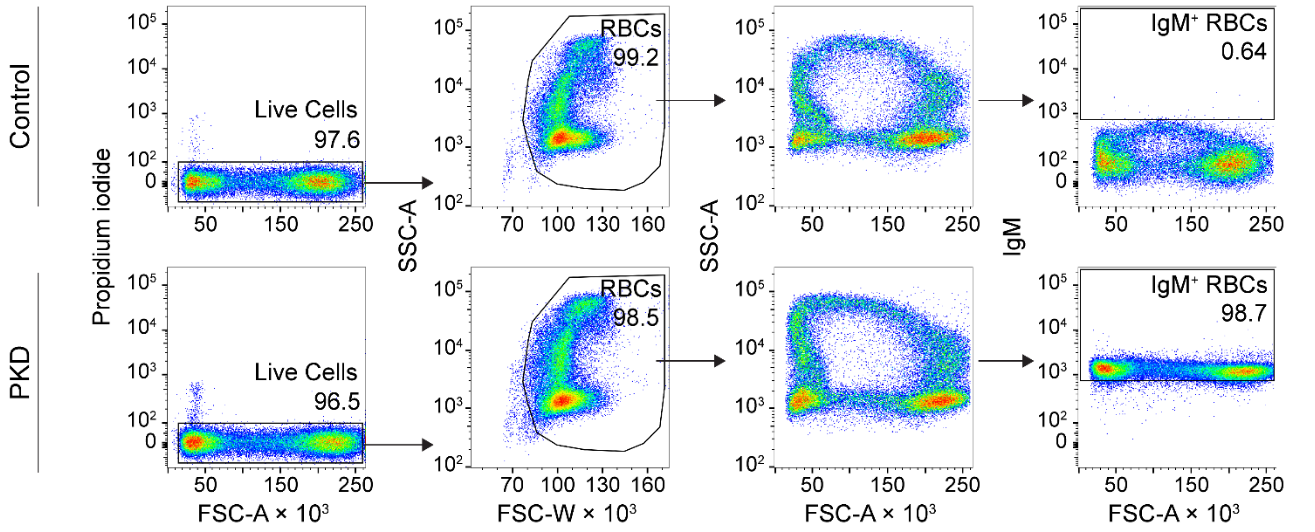

Supplementary Figure 8 | Propidium iodide was used to exclude dead cells from analyses or analyze the proportion of dead cells. These are representative gating strategies that show how we analyze the number of live IgM$^+$ red blood cells (RBCs) from either control fish in the top row or infected fish (PKD) in the bottom row. Numbers and labels refer to the proportion of cells a subpopulation makes up and the name of the gated subpopulation. E.g., only from infected fish (bottom right panel) can we detect a significant portion (98.7%) of RBCs staining positive with mAb 1.14. We used propidium iodide in combination with mAb 1.14 and anti-mouse IgG1-APC staining, and detected no spectral overlap between APC and propidium iodide. From left to right, we sequentially select subpopulations of propidium iodide-negative cells (representing live cells with intact membranes) followed by RBCs based on their side scatter area (SSC-A) versus forward scatter width (FSC-W) profile. The rightmost and final two plots show the resulting SSC-A versus FSC-A profile of the RBCs and the proportion of IgM$^+$ RBCs.
